# Superimposition of beams to vary shot size in gamma knife stereotactic radiosurgery

**DOI:** 10.1120/jacmp.v3i1.2588

**Published:** 2002-01-01

**Authors:** Morris I. Bank, Robert Timmerman

**Affiliations:** ^1^ Radiation Oncology Indiana University Medical Center 535 Barnhill Drive Indianapolis Indiana 46202

**Keywords:** gamma knife, radiation therapy, beam profiles

## Abstract

The Leksell Gamma Knife [Elekta Corp] uses helmets as collimators to produce four standard beam sizes. The nominal beam diameters are 18, 14, 8, and 4 mm. During computer treatment planning for gamma knife stereotactic radiosurgery, the size of the treated volume may differ from the standard beam sizes. To maintain conformality of the isodose curves to the treated volume, beam sizes may be superimposed during computer treatment planning to produce beam diameters that are intermediate to the standard beams. A study of superimposed gamma knife beams was performed to confirm the accuracy of this method and to verify the accuracy of the GammaPlan treatment planning computer. Superimposed beams were simulated on the Elekta treatment planning computer, GammaPlan, version 4.12, and tested by film measurements of beam profiles for single helmet sizes and superimposed shots with various beam weightings. The weighting for each beam size is varied to attain the beam size diameter desired. The beams were defined at the 50% isodose line. The profiles of the superimposed beams were obtained and compared with the single helmet shots. The uniformity of the resulting beams was measured. The results show a linear relationship between beam size and beam weighting for the superimposed beams. The film measurements confirm the computer calculations.

PACS number(s): 87.66.–a, 87.53.–j

## INTRODUCTION

The Leksell Gamma Knife [Elekta Corp] utilizes 201 intersecting Co‐60 beams to form the clinical beam. Primary collimators shape the initial beams and secondary collimators, mounted in external helmets, produce the clinically useful beam. Four helmets with different size collimators produce four standard beam sizes with nominal diameters of 18, 14, 8, and 4 mm.^1^ The gamma knife stereotactic radiosurgery treatment is planned from magnetic resonance imaging, cat scan, or angiography images on the Elekta GammaPlan computer planning system that models the four nominal beams. Treatment planning consists of placing “shots” or beams over the target volume. Multiple “shots” of the different helmet sizes are often used in order to attain conformality of an isodose volume over the outlined target volume.^2^ At our institution we attempt to maintain conformality with the 50% isodose volume positioned at the edge of the outlined target volume. During computer treatment planning, portions of the target volume's surface radius of curvature may not match the diameters of any of the four nominal beam apertures, resulting in less conformality than desired. To maintain conformality of the isodose curves to the target volume, different beam sizes may be superimposed during computer planning to produce beam diameters that are intermediate to the standard beam sizes and conform to the target volume. This technique is used for spherical lesions, as well as targets that have surface concavity or convexity. The computer calculations show that beam weighting for each shot size can be varied to attain the shot size diameter desired.

This study was performed to verify the accuracy of the GammaPlan computer calculated shot size diameters of superimposed beams. Superimposed beam shots with various beam weightings were calculated on the Elekta GammaPlan treatment planning computer. Film measurements of actual superimposed beams were obtained and compared with the computer calculations. The results verify the accuracy of the GammaPlan computer calculations.

## METHODS

### A. GammaPlan computer

Using the Elekta GammaPlan, version 4.12, planning computer beams, or “shots,” of different helmet sizes were superimposed on the same isocenter. The relative weighting of the beams was varied and the beam size at the 50% width in the transverse direction was measured using the tools of the GammaPlan The superposition of [18+14]‐, [14+8]‐, [8+4]‐, and [14+4]‐mm helmets were studied.

### B. Film measurements

To assess the accuracy of the GammaPlan computer modeling, images of the beams were obtained using a 16‐cm diameter spherical plastic phantom, supplied by Elekta mounted in our Model B gamma knife. A film cassette of the phantom allows mounting of film in the gamma knife beams. The phantom can be oriented in orthogonal planes, transverse, coronal, and sagittal. V‐film [Kodak] was placed in the film cassette of the phantom and exposed to the beam. The films were scanned in a Scanditronix RFA300 film scanner with RFA300Plus software version 5.2. The optical density (OD) was converted to dose using an OD to dose calibration curve obtained from exposing the V‐film to known doses at d=5 cm in a phantom from a Co‐60 unit. The profiles were obtained from the scans and the width of the 50% isodose was measured using the tools of the software. The beam size is defined at the 50% isodose width. The measured beam widths for the helmets in the three orthogonal planes are shown in Table I. Films were taken for superposition of beams for combinations of the 18‐, 14‐, 8‐, and 4‐mm helmets at weightings of 1:0, 3:1, 2:1, 1:2, 1:3, and 0:1.

**Table I:**
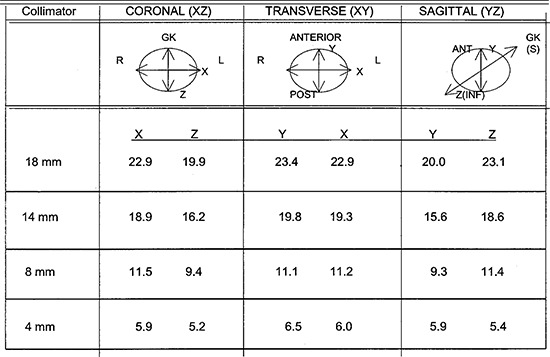
Gamma knife beam profiles, Model B, 50% width measured with V‐film in a plastic phantom.

## RESULTS

**Figure 1:**
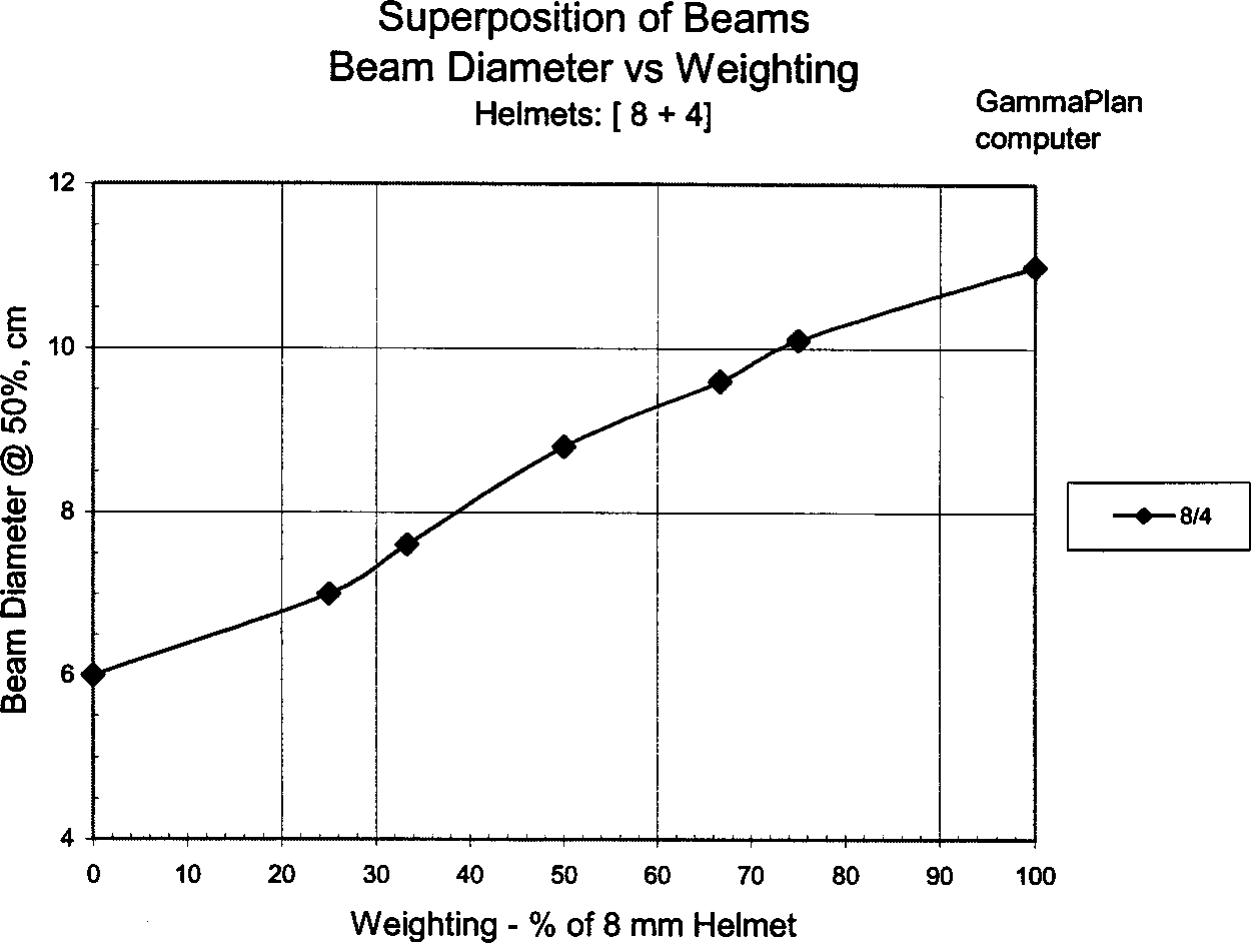
GammaPlan results for the superposition of 8‐ and 4‐mm helmets.

**Figure 2:**
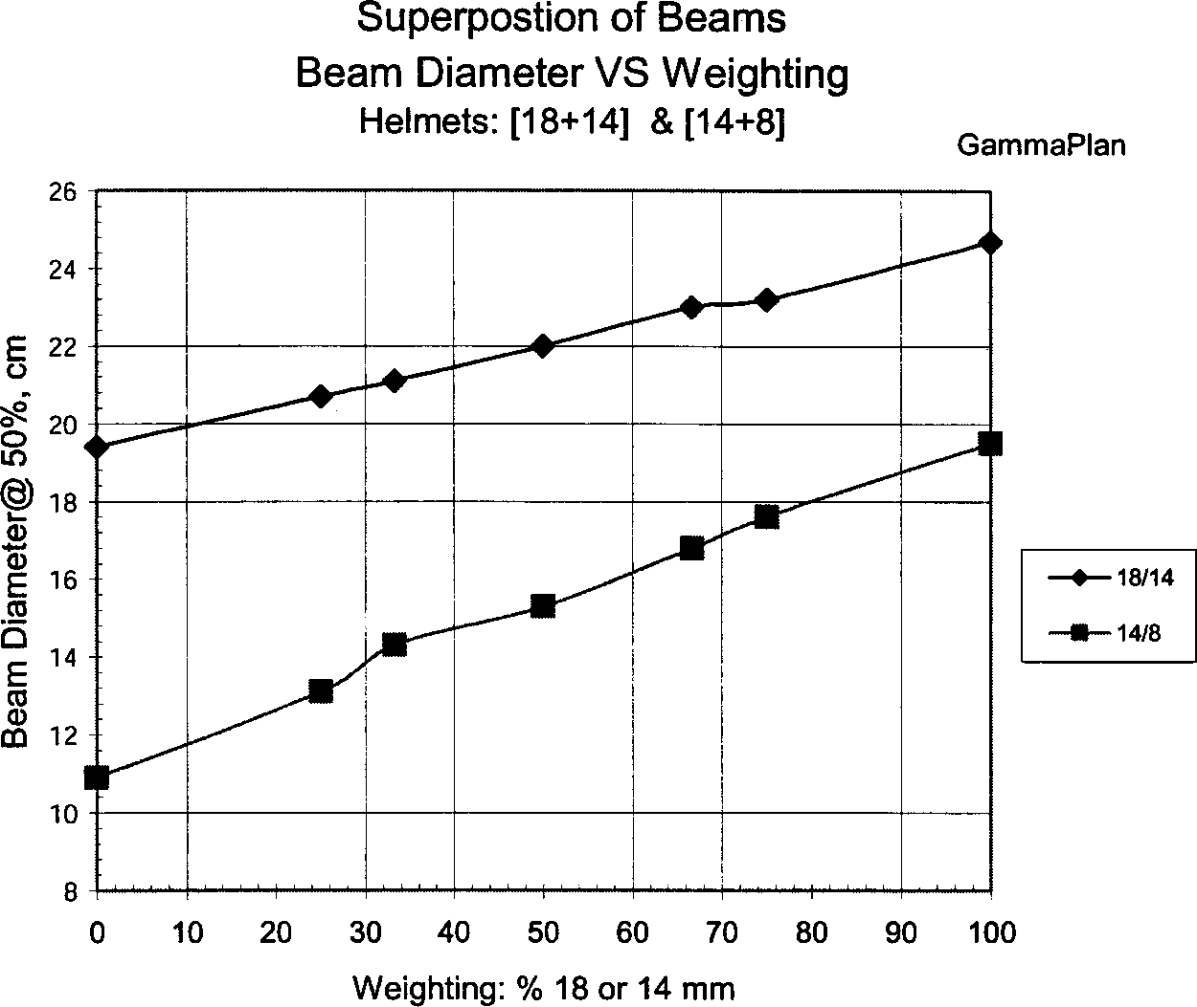
GammaPlan results for the superposition of 18‐ and 14‐ mm helmets, and 14‐ and 8‐mm helmets.

**Figure 3:**
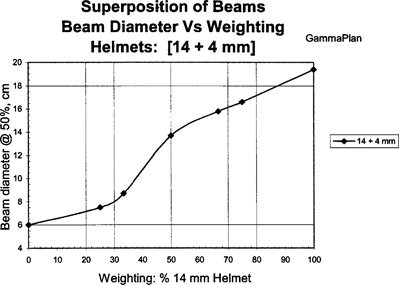
GammaPlan results for the superposition of 14‐ and 4‐mm helmets.

The GammaPlan computer generated shot sizes versus beam weighting are shown in Fig. 1 for superimposed [8+4]‐mm helmets, Fig. 2 for superimposed [18+14] and [14+8]‐mm helmets, and Fig. 3 for superimposed [14+4]‐mm helmet sizes. The beam widths were defined and measured at the 50% isodose line. The results show a linear relationship between shot size and beam weighting for the superimposed beams.

**Figure 4:**
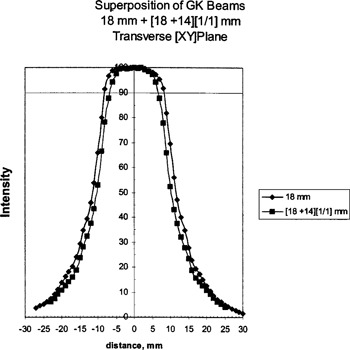
V‐film results for the 18‐mm helmet, and the superposition of 18‐ and 14‐mm helmets.

**Figure 5:**
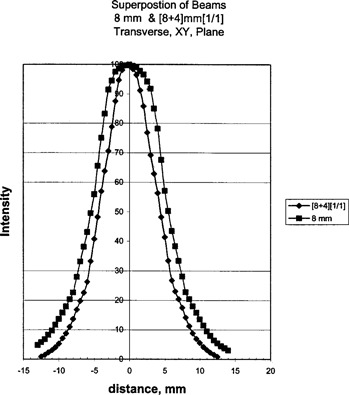
V‐film results for the 8‐mm helmet, and the superposition of 8‐ and 4‐mm helmets.

The film measurements of profiles of beams for 18‐mm helmets, and superimposed [18+14]‐mm helmets are shown in Fig. 4, with the 8‐mm helmet and the superimposed [8+4]‐mm helmets in Fig. 5. Both of these helmets are at 1/1 weighting. These results show a decrease in beam width when the helmets are superimposed.

**Figure 6:**
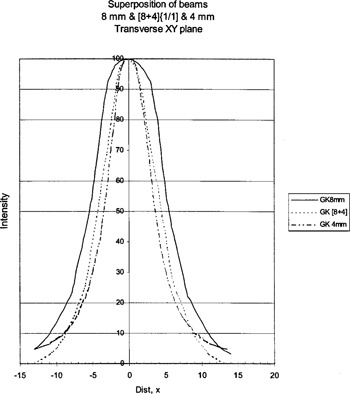
V‐film results for the 8‐mm helmet, 4‐mm helmet, and the superposition of 8‐ and 4‐mm helmets.

**Figure 7:**
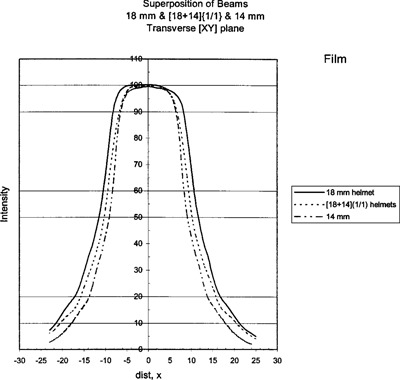
V‐film results for the 18‐mm helmet, 14‐mm helmet, and the superposition of 18‐ and 14‐mm helmets.

Figure 6 shows the 18‐, 14‐, and superimposed [18+14]‐mm profiles. Figure 7 shows the 8‐, 4‐, and superimposed [8+4]‐mm profiles. These plots show the superimposed shots are intermediate to the two helmets as predicted by the GammaPlan computer.

**Figure 8:**
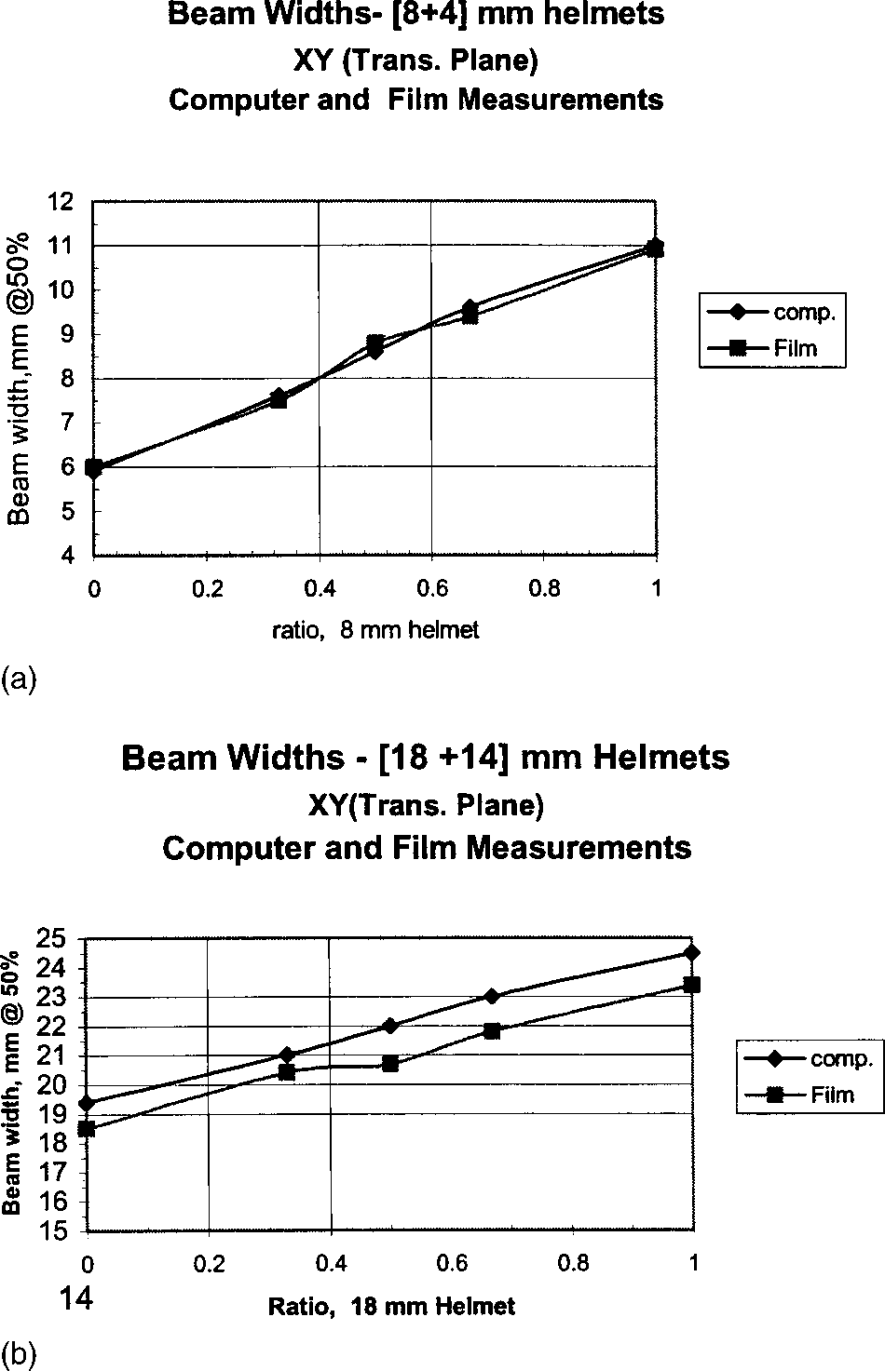
Measured beam widths from film for various weighting of superimposed gamma knife beams. (a) Superposition of 8‐ and 4‐mm helmets. (b) Superposition of 18‐ and 14‐mm helmets.

The measured beam widths for various weightings are shown in Fig. 8 for superposition of the [8+4]‐mm helmets (a), and the [18+14]‐mm helmets (b). The beam widths at the 50% isodose values do show a linear relationship similar to the computer results. The measured widths for the [18+14]‐mm helmets differ between the film and computer results, while the [8+4]‐mm helmets show good agreement.

The superimposed beams and the single helmet shots exhibit similar beam uniformity in their measured profiles. However, the superimposed beams exhibit less difference in width at the higher intensities.

## DISCUSSION

Maintaining conformality during treatment planning for Gamma Knife stereotactic radiosurgery is often limited by the beam size of the available helmets.^2^ This study demonstrates that the technique of superimposing beams can provide effective beam sizes intermediate to the available helmet sizes. Target sizes that are larger or smaller than the nominal beam sizes can be “fit” by the superimposed beams. It can be used for spherical lesions or targets that have surface concavities or convexities. Adjusting the weight ratio can achieve the conformal beam size. The study verifies the GammaPlan as accurately describing the superposition of the beams. The technique has clinical utility to improve conformality of the 50% isodose volume to the actual target volume. The method can also be used to move the maximum isodose volumes within the targeted volume by placing a smaller size shot in another position within the target volume.

## Conclusion

Superposition of gamma knife beams is a method of changing the effective beam size to obtain beam widths intermediate between two helmet sizes. This allows the planner to maintain conformality to a target volume. The film measurements confirm the computer calculations that show a linear relationship between the beam weighting ratio and effective beam size. The results verify the accuracy of the GammaPlan computer calculations.
